# What Determines the Perception of Fairness Regarding Household Division of Labor between Spouses?

**DOI:** 10.1371/journal.pone.0132608

**Published:** 2015-07-06

**Authors:** Mayumi Nakamura, Mito Akiyoshi

**Affiliations:** 1 Department of Economics, The University of Toyama, Toyama, Toyama, Japan; 2 Department of Sociology, Senshu University, Kawasaki, Kanagawa, Japan; Örebro University, SWEDEN

## Abstract

Married women often undertake a larger share of housework in many countries and yet they do not always perceive the inequitable division of household labor to be “unfair.” Several theories have been proposed to explain the pervasive perception of fairness that is incongruent with the observed inequity in household tasks. These theories include 1) economic resource theory, 2) time constraint theory, 3) gender value theory, and 4) relative deprivation theory. This paper re-examines these theories with newly available data collected on Japanese married women in 2014 in order to achieve a new understanding of the gendered nature of housework. It finds that social comparison with others is a key mechanism that explains women’s perception of fairness. The finding is compatible with relative deprivation theory. In addition to confirming the validity of the theory of relative deprivation, it further uncovers that a woman’s reference groups tend to be people with similar life circumstances rather than non-specific others. The perceived fairness is also found to contribute to the sense of overall happiness. The significant contribution of this paper is to explicate how this seeming contradiction of inequity in the division of housework and the perception of fairness endures.

## Introduction

Wives often do the majority of housework in dual-income households even when they work as much as their husbands in paid work [1]. The inequitable household division of labor is observed not only in those countries such as Japan where gender values are more conservative, but also in those countries such as North America and northern Europe where gender values are more egalitarian [2–5]. Yet, it is known that most women in those regions define their share of household chores as “fair” [5–7]. Why does this gap between perception and reality occur? What consequences does this perception have on their psychological well-being? These are the research questions to be investigated in this paper.

Japan is one of the most inequitable societies in this regard. An international comparison of ten countries on allocation of hours of dual-earner couples with young children shows that, in all ten countries (Japan, the United States, Belgium, German, France, Hungary, Finland, Sweden, the United Kingdom, and Norway), working wives with young children do more housework than their husbands [8]. And Japanese husbands spend by far the shortest time on housework (one hour and fifteen minutes) while husbands from other countries spend two to three hours.

In Japan, married women face major disincentives to staying in the labor force. The pay difference between sexes is large in Japan in comparison to other industrialized societies. This decreases the cost of not working for women. Long working hours make it difficult for women to retain their full-time job during their child-rearing years: 60% of working women leave their job after they give birth to their first child [9]. Further, Japan’s tax policy gives a substantial break to dependent spouses—i.e. spouses who earn 103 million yen (approximately 8,600 dollars) or less—in terms of income tax, national health insurance, and pension contributions [9, 10]. Married women sometimes deliberately limit their working hours in order to stay dependent for tax purposes because unless they make more than the break-even point as a non-dependent, they could end up with less take-home pay after taxes [9]. Women are thus often tracked into the role of primary homemaker and supplementary earner by institutional arrangements. The choice of limiting working hours is a classic case of “were they pushed or did they jump?”[11].

Yet women’s limited involvement with paid labor does not fully account for the housework inequity between genders. Here is an illustration: when the wife is not working outside the home, she spends on average 8.5 hours per day on housework, and her husband spends 50 minutes. When the wife is working outside the home for 35 hours or more per week, she spends on average 4.5 hours per day on housework, and her husband spends 55 minutes [8]. Even though this persistent labor inequity in Japanese households has been widely observed, there is scant understanding of the mechanism, a gap that this paper is addressing. Less is known about gender relations there, especially in comparison to North America and Europe, and a study of this situation will provide an extreme case against which future comparative research can benchmark.

It is known that women in advanced post-industrial societies do most of the household chores, and the majority of women find it fair [5–7]. A number of theories have been proposed to account for the perception of fairness against the backdrop of objective inequity observed in Japan and elsewhere. Yet as to Japan, the most plausible explanation remains to be established. After a review of relevant research, this paper will test existing theories to arrive at a better understanding of the gap between perception of fairness and reality of unequitable division of housework. Using regression methods to analyze a newly available dataset, we found that social comparison is the key mechanism explaining why Japanese wives keep pulling heavier weight in household work than their husbands.

## Literature Review

**Table 1** shows the time allocation of dual-earner couples with young children in the ten-country study mentioned previously. In all countries, women do most household chores, and this tendency is especially evident in Japan (**Table 1**).

**Table 1:** Average Time Allocation among Various Activities–Whole Week, Working Husbands and Wives with Child(ren) under 6 (for Japan and the U.S., under 5) (hours. minutes.).

												(Hour, Minutes)
			Japan	U.S.	Belgium	Germany	France	Hungary	Finland	Sweden	U.K.	Norway
Husband	Personal care activities		10.40	10.02	10.29	10.14	11.17	10.32	10.03	9.56	9.54	9.40
		Sleeping	7.52	8.11	7.59	7.50	8.26	8.05	8.12	7.48	8.09	7.47
		Grooming	2.48	1.52	2.30	2.23	2.51	2.27	1.51	2.08	1.45	1.53
	Work and travel related to work		8.01	6.17	5.04	4.58	5.29	5.23	5.48	5.11	5.56	4.57
	Housework and childcare		1.15	3.08	2.54	2.51	2.22	2.55	2.42	3.19	2.36	3.10
	Free time		2.33	4.11	3.49	4.26	3.37	3.58	4.04	3.58	3.48	4.43
Wife	Personal care activities		11.06	10.13	10.48	10.34	11.30	10.37	10.20	10.27	10.10	9.59
		Sleeping	7.57	8.22	8.23	8.06	8.40	8.23	8.21	8.08	8.17	8.02
		Grooming	3.09	1.50	2.24	2.28	2.50	2.14	1.58	2.19	1.53	1.58
	Work and travel related to work		3.37	4.59	3.32	2.18	3.47	3.38	3.38	2.42	3.17	2.37
	Housework and childcare		5.31	4.42	4.53	5.14	4.48	5.35	5.08	5.21	5.20	5.21
	Free time		2.18	3.40	3.17	4.15	2.46	3.05	3.22	3.53	3.22	4.44

Note. Data from “Survey on time use and leisure activities” [8]. Several categories such as “studying at school” and “other” are dropped by authors from the source to facilitate the reading of most time-consuming activities. Note that data is limited in comparability as the definition of activities and the timing of data collection vary across countries. See [8] for details.

Many previous studies on the division of housework have focused on wives’ sense of fairness [12–20]. Since husbands are the ones who benefit from dual income and the wife’s larger share of household chores, they are unlikely to find it unfair [12]. On the other hand, working wives are the ones whose perspectives are perplexing and need scrutiny, since while they bear the double shift of outside work and household labor, the majority of them still somehow find the division of household labor to be fair. Because this issue still requires explanation, we too focus on women’s perspectives.

Several theories have been proposed to explain the coexistence of inequity in housework and perceived fairness. These theories include: 1) economic resource theory; 2) time constraint theory; 3) gender value theory; and 4) relative deprivation theory [5]. Economic resource theory hypothesizes that the division of household labor and the associated perception of fairness depend on the balance of economic resource of the spouses [5]. A spouse with smaller economic resources (e.g., income) would engage in more household labor and find it fair [21–22]. In investigating why working wives perceive unfair division of housework as “fair” while they actually do twice as much housework as their husbands, Lennon and Rosenfeld claim that the lack of life options is the reason. Women “who have fewer alternatives to marriage and fewer economic resources are more likely to view a given division of housework as fair, while women with more alternatives view the same division as unjust” ([18], p.506). Moreover, those women who perceive their situation as “unfair” are more likely to suffer from poor psychological well-being.

According to time constraint theory, the balance of available time of the spouses influences the division of housework. A spouse with more spare time would engage in more household labor and find the arrangement fair [16]. Alternatively, gender value theory emphasizes that a wife with traditional gender role values would engage in more household labor and find it fair [15, 23–24]. Finally, relative deprivation theory holds that the division of household labor is a function of who a wife’s comparative referents are [5, 17].

Fuwa and Tsutsui showed that in those countries where women's average share of household labor is large, they do not perceive this objective inequity to be unfair. This is in contrast to countries where the division of household labor is more equitable. There, even a minor difference between men’s and women’s household working hours leads women to a perception of unfairness. Those authors conclude that the perceived national average is the comparative referent [5]. However, the mechanism through which the national average translates into individual women's perception of fairness is unclear [17]. There are few studies focusing on the link between the division of household labor of the couples and marital satisfaction, but they do not address comparative referent [20, 25–26].

Research on the perception of fairness further ignores the underlying affective dimension. Not only we do not know what explains the persistent and prevalent perception of fairness but also we are ignorant of the affective state associated with the perception. When wives perceive that the division of household work in their household is fair, are they happier than those who think they are doing more than they should? The lack of attention to affective dimensions is a long-standing one in sociology that needs to be addressed [27]. To be sure, the positive association between marriage and overall happiness is confirmed by numerous studies [28]. However, the literature on the marriage-happiness link is primarily concerned with the presence or absence of marriage and the quality of marriage does not get the attention it may warrant. It is plausible that a marriage where one spouse does a disproportionately large share of housework differs from a marriage where spouses share housekeeping responsibilities more evenly in terms of the level of happiness it achieves [20]. For a nuanced understanding of the positive effect of marriage on happiness, it is crucial to explore affective outcome of the perception of fairness. The current paper thus proposes to address the affective as well as the cognitive dimension of the division of housework between spouses. To this end, it incorporates the literature on happiness.

With her psychological experiment, Major showed that women tend to make a same-sex and same-job comparison regarding entitlement to their wages. Thus, women tend to compare themselves with someone of the same sex rather than their husbands [29]. In her review of previous studies, Thompson expanded on Major’s contention regarding distributive justice on wages to include division of household labor. She posited that women tend to compare their share of housework and wage work to that of other women rather than to their husbands, which explains why women can find the division of household chores "fair" when it is not a 50–50 split with the husband [19]. International comparative research suggests a similar dynamic in other countries as well. Women's labor force participation and gender values (at the national average) affect division of household labor [2]. Braun et al. showed that the average gender inequality (gender wage ratio) of a country affects women's perception of fairness in that country regarding division of household labor [24].

All of this points to social comparison as a key mechanism that leads to the perception of fairness, but details of the comparison process and affective consequences of comparison are not known. As we stated at the outset, a key tenet of this paper is that the theory of social comparison better explains observed outcomes such as the perception of fairness and happiness [30]. One notable proposition from the happiness literature is that utility derived from the ownership of a good depends on what others own [27, 30]. Relative gratification as well as relative deprivation are involved in social comparison [30–34]. It follows that those who think they are better off than their peers might think that their share of housework is “fair” even when the division of labor is inequitable and unfavorable to them. The notion of reference group suggests that satisfaction with relationship is associated with equity comparison with the partner and a reference group [35]. Existing studies on household division of labor remain somewhat vague about the mechanism of comparison, with some of them positing that women base their perception of fairness on a national average about which they are probably not aware. In contrast to this, we prefer to draw on theories of relative deprivation, relative gratification and reference group, and contend that a reference group can be the crucial comparator.

There is support for the reference-group hypothesis in other realms of women’s socialization and daily experience. The attitudes of close others are found to influence women’s gender role attitudes [35]. In particular, mothers are important in the development of daughters’ gender role attitudes. Mothers’ attitudes about motherhood and women’s role mediate the effects of their level of education and employment on daughters’ gender role attitudes [36]. It can be hypothesized that women who internalize traditional gender attitudes tend to see an inequitable division of labor as fair and just insofar as the arrangement conforms to existing norms.

Using micro-level data on Japanese women, we tested the aforementioned four theories (economic resource theory, time constraint theory, gender value theory, and relative deprivation theory) and concluded that relative deprivation can affect women’s perception of fairness of household division of labor, controlling for work hours, education, and their own gender values. We first confirmed that the perception of fairness is not determined solely by objective circumstances; i.e. wives’ share of housework vis-à-vis pertinent conditions such as income and working hours. Subsequently, we examined how the perception of fairness contributes to the level of happiness. It turns out that those who feel less unfairness in household division of labor have better overall sense of happiness. The perception of fairness, rather than actual share of housework, accounts for the level of happiness.

## Methods, Data, and Variables

The data used in the analysis was collected through a web-administered survey in March 2014, using monitors registered at Dentsu Macromill Insight, a research firm located in Tokyo (Hereafter we call this survey Women’s Work Life Survey).

The study was conducted to the highest ethical standards possible. The data collection involves a survey of human subjects and precautions were taken to protect subjects. The subjects were aware that their survey results were being used for current research. They were provided with a written informed consent statement in which the purpose of the survey was explained. The authors did not have access to identifying information about the participants of this survey. The survey administrator, Dentsu Macromill Insight, anonymized the data. Dentsu Macromill Insight is granted a “Privacy Mark,” an ethics approval certificate from the Japan Institute for Promotion of Digital Economy and Community (Their Privacy Mark Number is 12390012(07)). The Institutional Review Board is not established at research institutions with which the authors of this paper are affiliated and so no formal approval or waiver from an IRB was obtained.

The population (the theoretical target of the study from which a sample is drawn) is women whose ages range from 25 to 54, with a high school diploma or higher level of education, access to a personal computer and ability to answer a web survey. Since the compulsory education in Japan is 9 years, the respondents are slightly more educated than the general population (The Japanese compulsory schooling starts at age 6. The compulsory program is comprised of 6 years of elementary level and 3 years of middle school level. Most of middle school students continue onto high school. The percentage of people, aged 25 to 64 with at least high school diploma is 92.7% in 2011). With the survey being administered online, all respondents have basic computer skill to fill out the questionnaire online. The total number of collected cases was 2,344. Although the original data included both single and married women, this analysis uses only the 1,496 cases of married women, in order to test for hypotheses on division of labor between spouses.

Two sets of analysis were conducted with two types of dependent variables. The first analysis attempts to identify the key determinants of women’s perception of fairness in the household division of labor. Thus the dependent variable is the perception of fairness about division of household labor, measured on a five point scale. The question used for the dependent variable was, “Do you feel division of household labor between the spouses at your house is fair?” It asked respondents to rate on a five-point scale 1) very unfair to me, 2) somewhat unfair to me, 3) fair to both of us, 4) somewhat unfair to my husband, 5) very unfair to my husband. For regression analysis, the two categories “very unfair to my husband” and “somewhat unfair to my husband” were merged because these categories exhibit relatively fewer occurrences (6.1% and 0.7% respectively). “Very unfair to me” was coded as 1, “somewhat unfair to me,” as 2, “fair to both,” as 3, and “Very/somewhat unfair to my husband,” as 4.

We employ ordinary least square regression because it allows us to determine which variables affect the perception of fairness. The dependent variable is an ordinary variable with four categories. With respect to this type of dependent variable, some authors recommends the use of methods developed for categorical variables while others argue for the usefulness of ordinary least square analysis [37, 38]. We conducted both types of analyses and report the result of ordinary least square analysis after confirming that the results from the two types of methods do not contradict each other. Alternative analysis using multinomial logistic regression is provided in the supporting information that accompanies this paper [**S1 Table**]. Predicted relative log odds of selecting “unfair to wife” vs. “fair to both” and those of selecting “somewhat unfair to wife” vs. “fair to both” against others’ HHC based on Model S2 are shown in **S1 Fig.** [**S1 Fig**].

The second analysis, based upon the results of the first one, uses the sense of overall happiness as its dependent variable. The wording of the question is, “Taken all things together, would you say you are happy? The question was asked in ten point scale, ranging from 1 (very unhappy) to 10 (very happy). This way of measuring overall happiness draws on the General Social Survey [39]. The second analysis examines whether the perceived level of fairness translates to the sense of overall happiness controlling for other factors that can possibly influence happiness. We use ordinary least square regression because the dependent variable has ten values; methods for categorical data, even though theoretically possible, in reality produce too many empty cells and resulting models are unstable [38].

The independent variables used in the analysis are as follows:

- “Mother’s household chore (hereafter HHC)” refers to the respondent’s mother’s household share when respondent was 12 years old
- “Wife’s HHC”, referring to respondent’s own share of household chores
- “Others’ HHC”, referring to respondents’ perception of the wife’s share of household chores among couples in the general public with family settings similar to those of the respondent (e.g., work status and number of children)
- Years of education, to measure level of education
- Gender value
- Incomes of both husband and wife, in the form of logged class midpoint
- Age and squared age of the respondent
- “Work hours,” expressed as hours worked weekly.

Mother’s HHC was measured by the question: “How did your parents divide household labor between them when you were twelve years old?” It asked respondents to answer the percentage of household share for her mother and for her father. The percentage of mother’s share was used in the analysis. Wife’s HHC was measured with the question: “How do you divide household labor between you and your husband?” and asked the respondent to answer the percentage of household share for herself and her husband. The percentage for the respondent’s share was used in the analysis. The sum of wife’s and husband’s contribution is assumed to add up to 100 per cent since outsourcing housework is not common in Japanese households. Paid household aids constitute 0.1% of the total labor force. Japanese citizens are not allowed to hire non-Japanese helpers [40]. Other’s HHC was measured with the question: “How do couples in general public, who have similar living conditions (e.g., work status, number of children) as yourself, divide household labor between wife and husband? Please give us your rough estimate.” It asked respondents to answer the percentage of household share for wife and husband. The percentage for wife’s share was used in the analysis.

Gender value was measured by the question, “It is better for a husband to work outside, and for a wife to take care of the family” asking respondents to rate on five point scale (1 = agree to 5 = disagree). The gender value coding was reversed so that egalitarian values take smaller numeric values.

In addition, in the second analysis, the perception of fairness, the dependent variable in the first analysis, is included in independent variables to investigate whether the perceived level of fairness makes a difference in the level of overall happiness.

The first set of analysis is reported in Section “What Makes Women Perceive ‘Fairness’ in the Household Division of Labor?” The result of the second set of analysis is detailed in Section “Does the Perception of Fairness Increase Happiness?”

## Results

### What Makes Women Perceive “Fairness” in the Household Division of Labor?

Correlations among variables are summarized in **Table 2**. Mother’s HHC, wife’s HHC, and others’ HHC are found to be associated with the fairness variable although the size and statistical significance of their influence is to be assessed with regression analyses. **Table 3** indicates that 46% of the respondents deem the division of household labor unfair to them. 47% think that it is fair to both parties. About 7% say that it is unfair to their husbands.

**Table 2:** Correlation Matrix.

	1.	2.	3.	4.	5.	6.	7.	8.	9.	10.	11.	12.	13.
	Fairness	Happiness	Mother's	Wife's	Others'	Age	Number of	8. Wife's	Husband's	Wife's	Husband's	Gender	Wife's
			HHC	HHC	HHC		Children	income	income	education	education	value	work
1.	1												
2.	.270***	1											
3.	0.033	-.009	1										
4.	-0.355***	-.358	0.126***	1									
5.	0.285***	.141***	0.120***	0.163***	1								
6.	-0.116***	-.025	-0.007	0.044†	-0.059*	1							
7.	-0.116***	.106***	-0.009	-0.071*	-0.063*	0.222***	1						
8.	-0.086***	-.071**	-0.004	-0.163***	-0.163***	0.071**	-0.032	1					
9.	-0.040	.083**	0.022	0.099***	0.128***	0.214***	0.039	0.164***	1				
10.	0.060*	.055**	0.036	-0.012	0.089***	-0.095***	-0.106***	0.047†	0.149***	1			
11.	0.066**	.102***	0.049**	0.006	0.109***	-0.011	-0.076*	-0.036	0.205***	0.406***	1		
12.	-0.088***	-.058**	-0.016	-0.184*	-0.120***	0.043†	0.001	0.165***	-0.069**	0.048*	0.008	1	
13.	-0.024	-.104***	-0.048	-0.138***	-0.108**	-0.040	-0.100*	0.464***	-0.053	0.059†	0.028	0.183***	1
Mean	2.51	7.07	91.99	82.05	76.34	40.5	2.41	2.5	7.46	3.15	3.71	2.98	2.31
S.D.	0.79	2.05	14.573	14.95	13.22	7.84	1	2.3	3.27	1.21	1.61	1.12	1.22

Note. Note: Data from Women’s Work Life Survey. Pearson correlation coefficients.

*** p < .001.

** p < .01.

* p < .05.

†p < .10.

**Table 3:** Frequency Distribution of Categorical Variables (%).

Division of household	Very unfair to me	10.7
labor fairness	Somewhat unfair	35.3
	to me	
	fair to both of us	47.2
	Somewhat unfair	6.1
	to my husband	
	Very unfair	0.7
	to my husband	
Wife's income	No income	38.8
	1–100 million yen	30.8
	101–200	12.7
	201–300	6.2
	301–400	4.4
	401–500	2.4
	501–600	1.1
	601–700	0.5
	701–800	0.4
	801–900	0.3
	901–1000	0.1
	1001–1200	0.2
	1201 or more	0.1
	Don't know	1.9
Husband's income	No income	1.3
	1–100 million yen	1
	101–200	3
	201–300	9
	301–400	16.2
	401–500	16.5
	501–600	13.3
	601–700	9.4
	701–800	9
	801–900	6.3
	901–1000	3
	1001–1200	2
	1201–1500	1
	1501 or more	0.6
	Don't know	8.5
Wife's education	Senior high school	45.7
	or less	
	Vocational school	13.3
	Junior college	21.8
	University or	19.3
	graduate school	
Husband's	Senior high school	36.5
education	or less	
	Vocational	11.4
	Junior college	5.1
	University or	46.4
	graduate school	
Gender value:		
Wife should stay	Agree	9.2
home	Somwhat agree	25.7
	Neutral	33.5
	Somwhat disagree	21.1
	Disagree	10.6
Wife's work hours	1–20	14.9
	21–30	12.9
	31–40	13.9
	41–50	4.1
	51–60	1.1
	61 or more	0.2
	Various	0.7
	Not applicable	52.2
Number of children	0	22.6
	1	26.8
	2	38.8
	3	10.4
	4 or more	1.4

Note. Data from Women’s Work Life Survey. Percentages may not sum to 100 because of rounding. N = 1,496.

Descriptive statistics reveal some remarkable patterns regarding wives’ income. **Table 3** indicates that 39% of the respondents reported that they have no income. 31% make less than 1 million yen (8400 in US dollars). The concentration of wives’ income to the two lower brackets of income distribution is coterminous with the gender pay gap and the tax policy detailed in the Introduction. The financial position of women is one of the factors that affect the sense of fairness. **Table 2** shows that the sense of fairness is negatively associated with wife’s income (-.086, p <. 001). Combined with **Table 3**, **Table 2** suggests that although Japanese wives are in general not making as much contribution to their household income as their husbands are, perceived fairness decreases as their income increases. As discussed in the previous section, the four theories offer different implications of income for the perception of fairness. We thus incorporate wives’ as well as husbands’ income in statistical models to explore the validity of these theories.

The hypotheses to be tested are 1) Economic resource theory; 2) Time constraint theory; 3) Gender value theory; and 4) Relative deprivation theory. **Table 4** shows the results of the ordinary least square regression analyses on determinants of married women’s “perception of fairness” on household division of labor.

**Table 4:** Determinants of “Perception of Fairness” on Household Division of Labor.

	Model 1			Model 2		
	Coefficients		S.E.	Coefficients		S.E.
Constant	2.861	***	.498	3.056	***	.505
Mother's HHC	.002	*	.001	.002		.001
Wife's HHC	-.023	***	.001	-.023	***	.001
Others' HHC	.017	***	.001	.016	***	.001
Age	-.018		.022	-.015		.022
Age Squared	.000		.000	.000		.000
Number of Children	-.044	**	.017	-.052	**	.018
Wife's Education	.022	†	.012	.023		.012
Husband's Education	.005		.009	.006		.009
Wife's Income	-.021	**	.003	-.008	*	.003
Husband's Income	.013		.009	.005		.010
Gender value	.071	***	.015	.053	**	.016
Working Hours				-.009	***	.002
Adjusted R square	.319			.334		

Note: Data from Women’s Work Life Survey. Ordinary least square regression. Estimates and standard errors are obtained by regressing happiness on independent variables. Coding done by authors as described in text.

*** p < .001.

** p < .01.

* p < .05.

†p < .10.

Model 1 tests for 1) economic resource theory, 3) gender value theory, and 4) relative deprivation theory. The result partially supports economic resource theory, but it qualifies this theory in one crucial respect. Wife’s income is statistically significant, but husband’s is not. When wife’s income is larger, wives feel their division of household labor as unfair to themselves. Economic resource matters, yet it is wife’s own economic resource that affects her perception. The result indicates that the fairness evaluation is based on wife’s own contribution to household income, not on a proportional contribution by each spouse.

Model 1 also supports gender value theory and relative deprivation theory. When wife’s gender value is more egalitarian, they tend to find their division of household labor as unfair. Respondent’s own share of housework and others’ share are statistically significant. The opposing signs of the coefficients of respondent’s own share and others’ share indicate that the respondents engage in comparison. As the respondent’s own share of housework increases, perceived fairness decreases while the share of others is positively associated with the sense of fairness.

Model 2 tests for all four hypotheses, including 2) time constraint theory. Model 2 reveals that, once we test for time constraint theory, controlling for respondents’ work hours, the effect of wife’s income decreases. The effect of gender values is also reduced and its significance is reduced (p < .05 in Model 2 vs. P < .001 in Model 1). The effect of wife’s work hours is significant, suggesting that the longer wives work, the more unfair they find the division of household labor is. The number of children remains significant when respondent’s work hours are controlled. Together, these two variables capture the relevance of time constraints. As for relative deprivation theory, mother’s HHC is not significant in Model 2. Wife’s HHC and others’ HHC stay significant.

**Fig 1:**
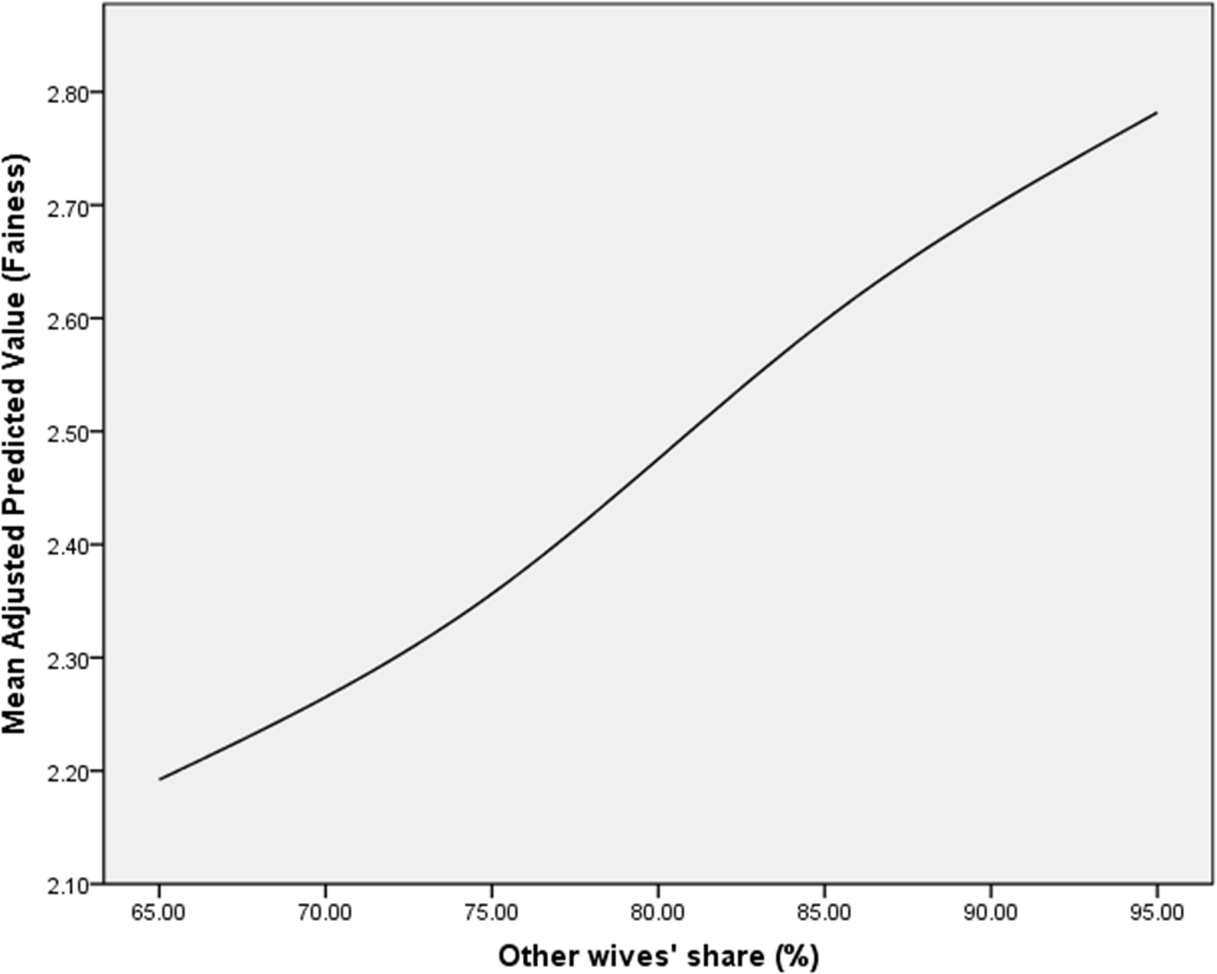
Perception of Other Wives’ Share of HHC on Woman’s Perception of Fairness around here.

In sum, ordinary least square regression analyses demonstrate that time constraint theory and relative deprivation theory are supported by the data. There is some evidence in support of economic resource theory and gender value theory. Different data and different analyses may bring them back in the picture. In the context of the analyses presented here, however, the most remarkable finding is the influence of comparison with others. The effect of others’ HHC indicates that relative gratification, or more precisely, relative consolation is at work. Fig 1 shows that as the share of housework ascribed to others by the respondent increases, the latter’s perceived fairness increases, holding her own housework share constant (Fig 1). In other words, to the extent a respondent believes that other wives are doing a large share of housework, her perception of fairness is preserved. Therefore, relative deprivation theory is valid in principle, but the more generic “social comparison” may be a more appropriate label.

### Does The Perception of Fairness Increase Happiness?

The analysis in the previous section has shown that the perception of fairness in household division of labor is a function of women’s view on the circumstances of others. The proportions of household labor borne by “others like me” were both statistically significant. Of course, the actual share of household labor done by others may not be accessible to women’s scrutiny. Women might have a good grasp of how much household work their mother had been doing in the family they grew up in or they might discuss how much they are doing with their friends and learn how other couples split the responsibilities, but even such recollections and indirect inputs may not be accurate as a description of reality. This inaccessibility of the circumstances of others does not result in the trivialization of social comparison. Our analysis is based on the respondents’ best guess of shares of the others. It rather shows that even when accurate data is not available, women make an inference and that their educated guess does influence their perceived level of fairness.

Our subsequent question is therefore how the perception of fairness in turn affects married women’s sense of happiness. Previous studies claim that those women who find their division of household work as unfair experience poor psychological well-being [18]. Do those who perceive more fairness tend to be happier in our sample, controlling for variables such as work hours, income, and education? The simple pairwise correlation shown in Fig 1 indicates that there is an association between the perception of fairness and happiness, but the correlation by definition does not take the effects of other variables into account. The correlation between fairness and happiness is .270 and it is significant at .001 level. Therefore the direction of association is consistent with our conjecture that the higher level of fairness leads to the higher level of happiness. In order to better understand the effect of the perception of fairness on happiness, the second set of models is tested to account for self-reported levels of happiness with the independent variables examined in the previous section and the fairness variable.

**Table 5** shows the results of ordinary least square regression analyses on married women’s happiness. Model 3 uses all the independent variables used in Model 2. The result of Model 3 shows that when the perception of fairness is not controlled for, wife’s share of housework and others’ share appear statistically significant. Note that the effect of wife’s share is negative while the effect of others’ share is positive. The direction of associations is compatible with the relative deprivation theory as discussed with respect to Models 1 and 2. As a wife assumes a greater share of the housework, she becomes less happy. As others with similar life circumstances assume a greater share, she becomes more happy.

**Table 5:** Determinants of Sense of Happiness.

	Model 3			Model 4		
	Coefficients		S.E.	Coefficients		S.E.
Constant	7.753	***	1.468	8.086	***	1.433
Mother's HHC	.003		.003	.002		.003
Wife's HHC	-.014	***	.003	-.001		.004
Others' HHC	.015	***	.004	.006		.004
Age	-.084		.065	-.075		.064
Age Squared	.001		.001	.001		.001
Number of Children	-.016		.051	.018		.050
Wife's Education	-.034		.034	-.047		.034
Husband's Education	.101	***	.025	.093	***	.025
Wife's Income	-.004		.010	.000		.010
Husband's Income	.026		.029	.013		.028
Gender value	.060		.045	.013		.045
Working Hours	-.004		.005	.000		.004
Unfair to Me (Wife)				-1.616	***	.184
Somewhat Unfair to me				-.617	***	.115
Very Unfair/Somewhat Unfair to My Husband				-.149		.202
Fair to both of us				0.000^a^		
Adjusted R square	0.054			0.105		

Note: Data from Women’s Work Life Survey. Ordinary least square regression. Estimates and standard errors are obtained by regressing happiness on independent variables. Coding done by authors as described in text.

*** p < .001.

** p < .01.

* p < .05.

†p < .10.

The effect of relative deprivation on happiness is mediated by the perception of fairness. Model 4 adds each level of fairness as a dummy. “Fair to both” is treated as a baseline category. Model 4 shows that the levels “very unfair to me” and “somewhat unfair to me” are statistically significant. “Very/somewhat unfair to husband,” a minority category, is not significantly different from the baseline category. Married women are less happy when they feel that the division of household labor is “very unfair to me” or “somewhat unfair to me.”

**Fig 2:**
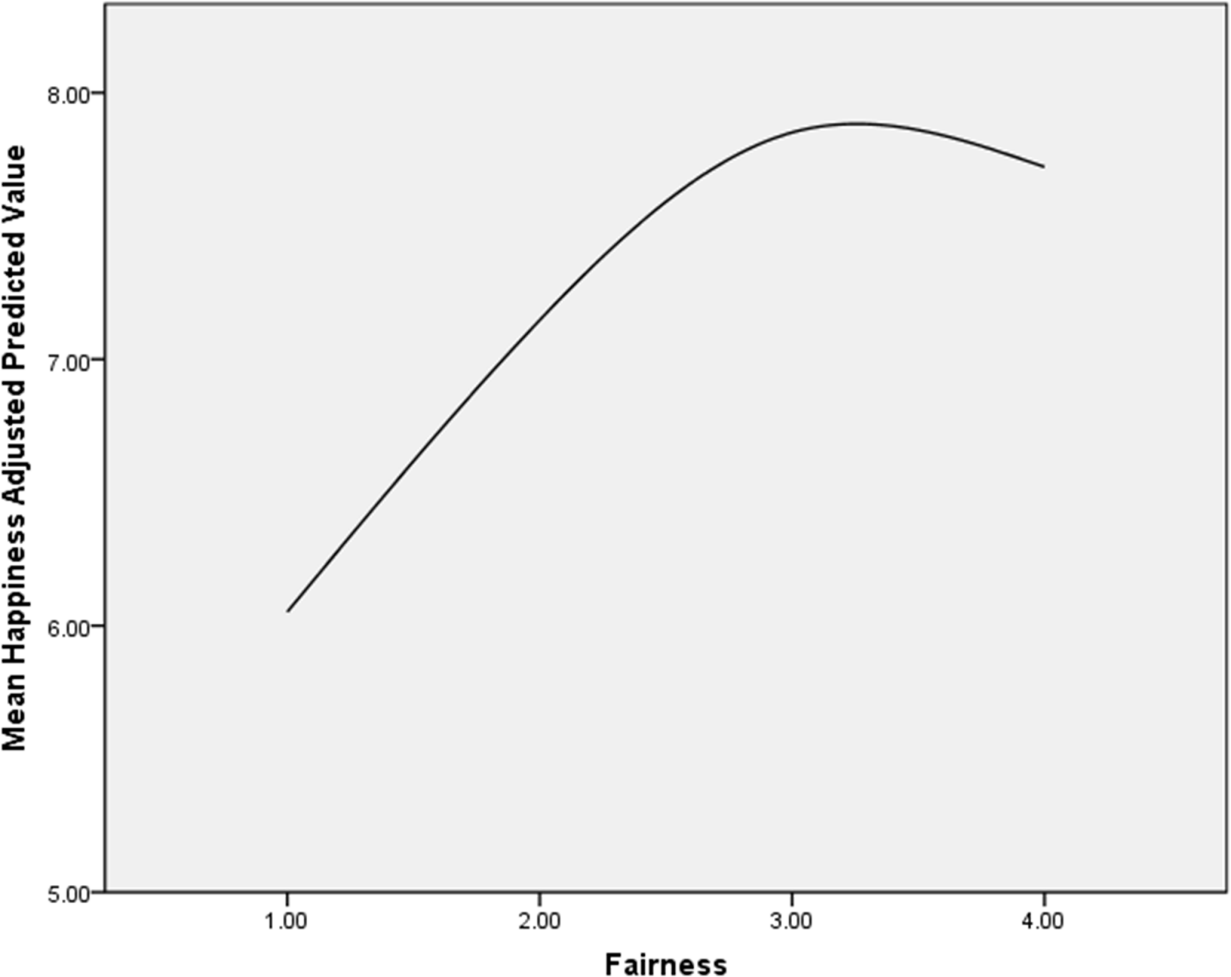
Predicted Adjusted Mean of Happiness and Woman’s Perception of Fairness around here.

Fig 2. plots predicted levels of happiness against the level of perceived fairness. It indicates that those who think that their arrangement is “fair to both” is the happiest.

## Discussion

This paper has investigated why married women in Japan often perceive their share of housework to be fair even when they assume a disproportionately larger share than their husbands. It reexamined the four predominant explanatory frameworks: 1) economic resource theory, 2) time constraint theory, 3) gender value theory, and 4) relative deprivation theory. Survey data collected in 2014 are employed to test these theories. Regression analyses revealed that wife’s income and gender values have limited effect. On the other hand, wife’s work hours and perceptions of others’ share were found to have substantial influence on the perception of fairness. In plain terms, wives do not necessarily perceive their share of housework to be unfair when they do more housework than their *spouses*. Rather, they sense unfairness when they think they are doing more than *others with similar life circumstances*. Furthermore, the perception of fairness increases general happiness. The most intriguing finding is the power of reference group. When we control for gender values, the division of household labor of the reference group does affect respondents’ perception of fairness. This suggests that even when married women have not internalized traditional gender values, they still accept an unbalanced division of household labor as fair, if they believe that people around them with similar life settings have an even more inequitable domestic situation. Thus, relative deprivation theory has good explanatory power. The theory lends support to the centrality of social comparison in determining the level of happiness. Even more remarkably, while Easterlin thinks that social comparison is weak in family life, our results suggest that the private nature of family life does not keep people from judging their circumstances in relation to those of others. Within the domain of family life, the division of labor may be a sub-domain where social comparison is quite prevalent whereas life events such as marriage and divorce may be relatively immune to social comparison. The varied degrees of social comparison with regard to various components of a domain is acknowledged by Easterlin and others [30] Women evaluate their share of household labor against the going rate for someone like themselves. Moreover, the perception of fairness in household division of labor actually affects married women’s sense of overall happiness.

The results paint a somewhat ironic picture. If a woman perceives that other wives in similar life settings are bearing with a severely unbalanced household division of labor, then she sees her own unbalanced division of labor as fair and that state of affairs contributes to her overall happiness. This finding of the link between the perception of fairness and happiness corroborates findings from previous studies done in other societies [18, 20]. Different levels of women’s labor force involvement notwithstanding, the process of social comparison is prevalent across societies in determining the perception of fairness. By not knowing there are couples with better-balanced household division of labor, those women actually manage to be happier, even when their division of labor is unbalanced.

Although the current research shows the robust link between the perception of fairness and others’ housework share, further research is needed as to the comparative processes through which women form judgments about their share of housework. How do women form their perception of other women’s share of household chores? Do wives compare notes with their married friends? How accurate are their evaluations? These questions merit future research.

We have argued here that the fact of inequity does not lead to the perception of unfairness. Women sense unfairness only when they believe that others with similar life circumstances are faring better than themselves. In a similar vein, the amount of housework itself does not determine women’s happiness, but the perception of fairness does. Whether illusory or not, by perceiving that other wives are faring equally poorly in household division of labor or worse, women do far more household work than their husbands do and yet stay happy.

## Supporting Information

S1 Dataset Women’s Work Life Survey. (ZIP)

S1 Fig The effect of others’ HHC. (TIFF)

S1 Table Determinants of “perception of fairness” on household division of labor (multinomial logistic regression). (DOCX)
