# Supplementary figures and images for: What Determines the Perception of Fairness Regarding Household Division of Labor between Spouses?
Source: PLoS One. 2015 Jul 6;10(7):e0132608. doi: 10.1371/journal.pone.0132608 (PMC4493123; doi:10.1371/journal.pone.0132608)

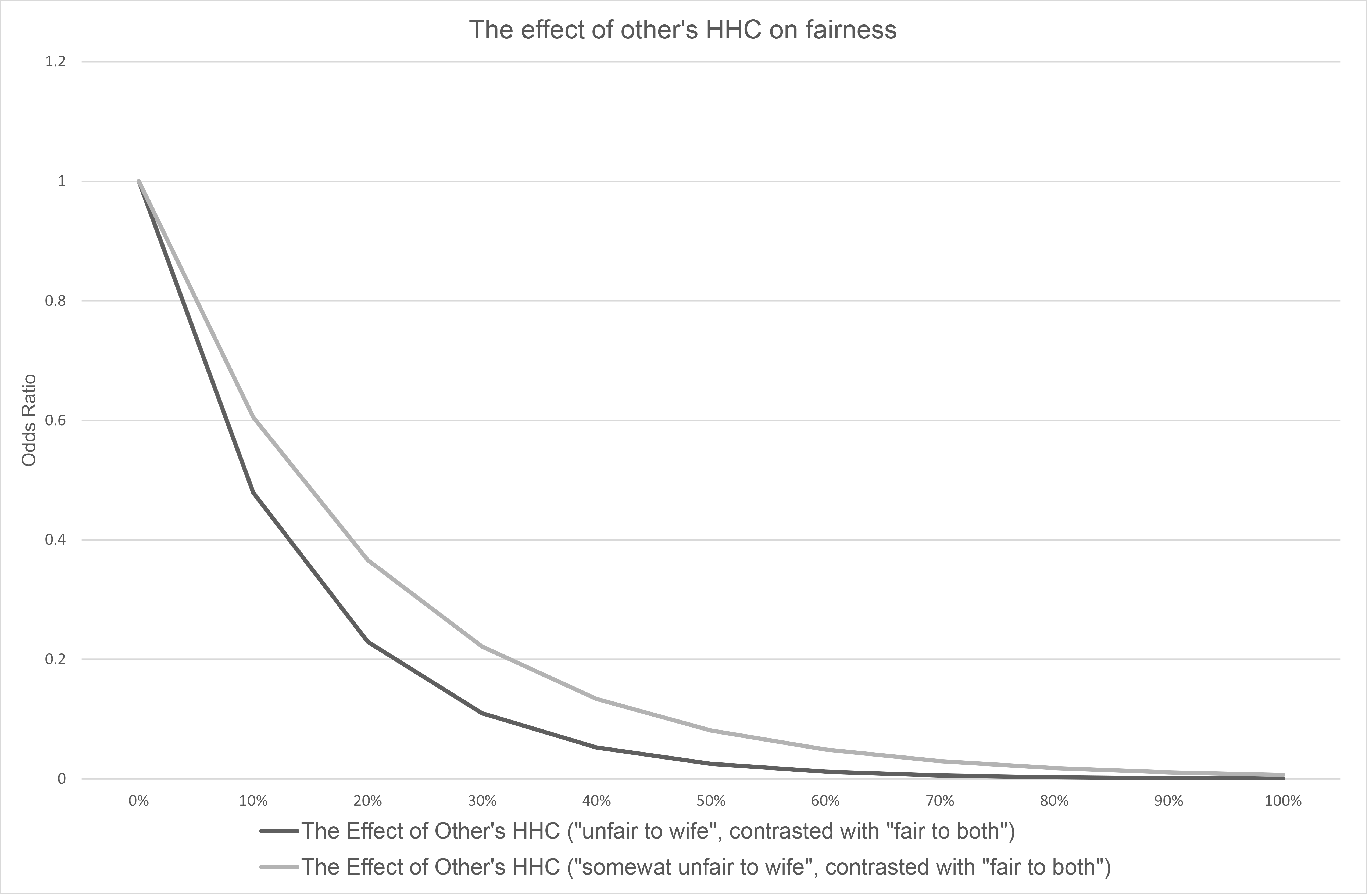

Supplement: S1 Fig — (TIFF) [file pone.0132608.s002.tiff]
