# Supplementary material for: What Determines the Perception of Fairness Regarding Household Division of Labor between Spouses?
Source: PLoS One. 2015 Jul 6;10(7):e0132608. doi: 10.1371/journal.pone.0132608 (PMC4493123; doi:10.1371/journal.pone.0132608)
Supplement: S1 Table — (DOCX) [file pone.0132608.s003.docx]

S1 Table. Determinants of “Perception of Fairness” on Household Division of Labor (Multinomial logistic regression).

|  |  | Model S1 | | | | Model S2 | | | |
| --- | --- | --- | --- | --- | --- | --- | --- | --- | --- |
|  |  | B | S.E. |  | Exp (B) | B | S.E. |  | Exp (B) |
| unfair to wife | Constant | -8.730 | 3.358 | ** |  | -9.975 | 3.511 | ** |  |
|  | Mother's HHC | -.009 | .007 |  | .991 | -.004 | .007 |  | .996 |
|  | Wife's HHC | .141 | .012 | *** | 1.152 | .150 | .013 | *** | 1.162 |
|  | Other's HHC | -.075 | .008 | *** | .928 | -.074 | .009 | *** | .929 |
|  | Age | .170 | .145 |  | 1.185 | .137 | .150 |  | 1.146 |
|  | Age Squared | -.002 | .002 |  | .998 | -.001 | .002 |  | .999 |
|  | Number of Children | .282 | .106 | ** | 1.326 | .351 | .113 | ** | 1.420 |
|  | Wife's Education | .026 | .074 |  | 1.026 | .001 | .077 |  | 1.001 |
|  | Husband's Education | -.097 | .052 | † | .908 | -.088 | .055 |  | .916 |
|  | Wife's Income | .090 | .017 | *** | 1.094 | .021 | .024 |  | 1.021 |
|  | Husband's Income | -.124 | .048 | * | .884 | -.098 | .054 | † | .906 |
|  | Gender Value | -.413 | .095 | *** | .662 | -.335 | .099 | ** | .716 |
|  | Working Hours |  |  |  |  | .044 | .010 | *** | 1.045 |
| somewhat unfair to wife | Constant | .208 | 1.930 |  |  | -.404 | 2.010 |  |  |
|  | Mother's HHC | -.002 | .005 |  | .998 | .000 | .005 |  | 1.000 |
|  | Wife's HHC | .057 | .005 | *** | 1.058 | .059 | .006 | *** | 1.061 |
|  | Other's HHC | -.053 | .005 | *** | .949 | -.051 | .006 | *** | .951 |
|  | Age | .045 | .085 |  | 1.046 | .048 | .087 |  | 1.049 |
|  | Age Squared | .000 | .001 |  | 1.000 | .000 | .001 |  | 1.000 |
|  | Number of Children | .086 | .066 |  | 1.090 | .119 | .068 | † | 1.127 |
|  | Wife's Education | -.117 | .045 | ** | .890 | -.120 | .046 | ** | .887 |
|  | Husband's Education | .019 | .033 |  | 1.019 | .011 | .035 |  | 1.011 |
|  | Wife's Income | .065 | .010 | *** | 1.067 | .038 | .014 | ** | 1.039 |
|  | Husband's Income | -.055 | .042 |  | .946 | -.052 | .046 |  | .950 |
|  | Gender Value | -.254 | .060 | *** | .775 | -.226 | .062 | *** | .798 |
|  | Working Hours |  |  |  |  | .020 | .006 | ** | 1.020 |
|  |  |  |  |  |  |  |  |  |  |

S1 Table. (continued.)

|  |  |  |  |  |  |  |  |  |  |
| --- | --- | --- | --- | --- | --- | --- | --- | --- | --- |
|  |  | Model S1 | | | | Model S2 | | | |
|  |  | B | S.E. |  | Exp (B) | B | S.E. |  | Exp (B) |
| unfair to husband or somewhat unfair to husband | Constant | .606 | 3.268 |  |  | 1.361 | 3.372 |  |  |
|  | Mother's HHC | .019 | .010 | † | 1.019 | .023 | .010 | * | 1.024 |
|  | Wife's HHC | -.058 | .008 | *** | .943 | -.059 | .008 | *** | .943 |
|  | Other's HHC | .043 | .011 | *** | 1.044 | .041 | .011 | *** | 1.042 |
|  | Age | -.093 | .144 |  | .911 | -.105 | .148 |  | .901 |
|  | Age Squared | .001 | .002 |  | 1.001 | .001 | .002 |  | 1.001 |
|  | Number of Children | -.098 | .120 |  | .907 | -.098 | .121 |  | .907 |
|  | Wife's Education | .089 | .079 |  | 1.093 | .068 | .081 |  | 1.070 |
|  | Husband's Education | -.041 | .060 |  | .960 | -.041 | .061 |  | .960 |
|  | Wife's Income | -.054 | .018 | ** | .947 | -.008 | .023 |  | .992 |
|  | Husband's Income | -.091 | .057 |  | .913 | -.112 | .062 | † | .894 |
|  | Gender Value | -.094 | .105 |  | .910 | -.180 | .110 |  | .835 |
|  | Working Hours |  |  |  |  | -.032 | .012 | ** | .968 |
| Psuedo | Cox & Snell | .347 |  |  |  | .363 |  |  |  |
| R square | Nagelkerke | .386 |  |  |  | .404 |  |  |  |
|  | McFadden | .187 |  |  |  | .198 |  |  |  |
|  |  |  |  |  |  |  |  |  |  |

Note: Data from Women’s Work Life Survey. Multinomial logistic regression. Alternative models to Model 1 and Model 2 in text. Estimates and standard errors are obtained by regressing fairness on independent variables. Coding done by authors as described in text. Baseline category is “fair to both.”

*** p<.001.

** p<.01.

* p<.05.

†p<.10.
